# Partial Dislocation of the Fourth Cervical Vertebra Due to Muscular Action

**Published:** 1879-08

**Authors:** John A. Wyett


### PARTIAL DISLOCATION OF THE FOURTH CERVICAL VERTEBRA DUE TO MUSCULAR ACTION.

BY JOHN A. WYETT, M. D.

On the morning of March 7 I was summoned to see a lady, who, I was told, had injured her neck. The history of the accident was as follows: In the act of bathing, while standing with the neck twisted, (the face being turned sharply to the left) she had lifted the right forearm and hand over the right shoulder, and was sponging herself between the scapula. While in this position she was seized with sudden and intense pain in the neck, more especially the right side. On arriving about thirty minutes after the accident, I found her suffering intensely; the neck was twisted to the left and immovable, and the face turned and looking over the left shoulder. Her left hand was grasping the right side of the neck over the fourth cervical articular processes. She complained that she could scarcely breathe, and that there was a painful numbness running down the right arm. On running my finger down along the processes of this side, I found there was an intense pain on pressure at the junction of the right articular processes of the fourth and fifth vertebra. Seizing the head I carefully attempted to rotate it to the right, but the entire body turned with it. Feeling confident that there was a dislocation forwards of the fourth articular process of the right side, upon the fifth, I seized the head from behind, on both sides, placing each hand with the thumb under the occiput, and the fingers under the jaw and chin, and turned the head slightly to the left, then made strong extension and rotated to the right. The head turned into its position without any trouble, and the pain instantly ceased. I moulded a shellac splint on the right side of the neck, and over the shoulder of the same side, and threw a figure of 8 roller around this shoulder and the neck. During the next two days there was considerable pain in the right arm and side, and along the track of the cord, which was relieved by morphine.

The patient recovered fully in a week, and has not since suffered. It is now more than three months since the accident. Dislocation of a vertebra, without fracture, is in itself a rare accident, and a simple displacement by muscular contraction has, as far as I am informed, not been reported. I am fortified in the correctness of the diagnosis in my case by the following facts:

1. There was complete fixation and immobility of the neck, which was relieved by the successful reduction.

2. Interference with respiration, showing that the filaments of the phrenic nerve were pressed upon. Pain in the arm, due to pressure on those filaments of the fifth nerve escaping from the fourth intervertebral foramen, which join the brachial plexus. Pain in the track of the cord, due to the slight pressure it received from an incomplete dislocation of the body of the vertebra. Entire disappearance of these symptoms at the moment of reduction.

3. That there was no fracture, was evident from the absence of crepitus and the rapid recovery. The symptoms could not have resulted from rupture of muscle or tendon, because it would not have rendered the neck immovable, nor would the pain have disappeared so rapidly in case of rupture, where there would have been more extravasation and consequently more material for absorption.—*The Hospital Gazette.*.

